# Effective Connectivity in the Human Brain for Sour Taste, Retronasal Smell, and Combined Flavour

**DOI:** 10.3390/foods10092034

**Published:** 2021-08-29

**Authors:** Justin Long Kiu Suen, Andy Wai Kan Yeung, Ed X. Wu, Wai Keung Leung, Hiroki C. Tanabe, Tazuko K. Goto

**Affiliations:** 1 Faculty of Dentistry, The University of Hong Kong, Hong Kong, China; justin.suen@novusls.com (J.L.K.S.); ndyeung@hku.hk (A.W.K.Y.); ewkleung@hku.hk (W.K.L.); 2 Department of Oral and Maxillofacial Radiology, Tokyo Dental College, 2-9-18, Kanda-Misakicho, Chiyoda-ku, Tokyo 101-0061, Japan; 3 Department of Electrical and Electronic Engineering, Faculty of Engineering, The University of Hong Kong, Hong Kong, China; ewu@eee.hku.hk; 4 Department of Cognitive and Psychological Sciences, Graduate School of Informatics, Nagoya University, Furo-cho, Chikusa-ku, Nagoya 464-8601, Japan; htanabe@i.nagoya-u.ac.jp; 5 Tokyo Dental College Research Branding Project, Tokyo Dental College, 2-9-18, Kanda-Misakicho, Chiyoda-ku, Tokyo 101-0061, Japan

**Keywords:** fMRI, insula of Reil, limbic system, neural network models, smell, taste

## Abstract

The anterior insula and rolandic operculum are key regions for flavour perception in the human brain; however, it is unclear how taste and congruent retronasal smell are perceived as flavours. The multisensory integration required for sour flavour perception has rarely been studied; therefore, we investigated the brain responses to taste and smell in the sour flavour-processing network in 35 young healthy adults. We aimed to characterise the brain response to three stimulations applied in the oral cavity—sour taste, retronasal smell of mango, and combined flavour of both—using functional magnetic resonance imaging. Effective connectivity of the flavour-processing network and modulatory effect from taste and smell were analysed. Flavour stimulation activated middle insula and olfactory tubercle (primary taste and olfactory cortices, respectively); anterior insula and rolandic operculum, which are associated with multisensory integration; and ventrolateral prefrontal cortex, a secondary cortex for flavour perception. Dynamic causal modelling demonstrated that neural taste and smell signals were integrated at anterior insula and rolandic operculum. These findings elucidated how neural signals triggered by sour taste and smell presented in liquid form interact in the brain, which may underpin the neurobiology of food appreciation. Our study thus demonstrated the integration and synergy of taste and smell.

## 1. Introduction

Flavour is a food-related perception that requires integration of different sensory systems in the brain. Perceived flavour (quality and intensity) affects consumers’ feelings (pleasantness and satiety), which are highly associated with appetite, ultimately affecting human health and quality of life. Flavour perception is part of daily life; however, the mechanisms underlying flavour perception have not yet been elucidated.

Following oral stimulation by liquid tastants or food, brain activation is commonly observed in the insula, operculum, thalamus, and orbitofrontal cortex (OFC) [1,2,3,4]. Past human brain imaging studies have focused on the responses to sweet, salty, and umami (a savoury taste from glutamate) tastes due to their direct influence on health or their general gustatory preferences. However, sour food is also relevant for human health. For example, lactic acid from sour milk enhances the immune system [5]. Anthocyanin, commonly found in sour cherries or other fruits, is characterised by strong antioxidant and anti-inflammatory properties [6,7]. Experiments on rats and macaque monkeys have demonstrated the activation of the gustatory cortex (the insula) and prefrontal cortex by pure sour taste [8,9]. Meanwhile, human neuroimaging studies usually focused on beer and wine, acidic beverages with bitterness [10,11,12]. However, few studies have examined brain activation by sour tastants. In a positron-emission tomography (PET) study with 10 participants, Small et al. [13] observed that the cortical representation of sour taste was mainly concentrated in the frontal cortex. A functional magnetic resonance imaging (fMRI) study with six participants by Schoenfeld et al. [14] observed activation in the ventral insula. Another fMRI study with 18 participants by Haase et al. [15] observed activation mainly in the frontal cortex, insula, thalamus, and caudate. Crouzet et al. [16] reported neural responses associated with sour taste from 16 participants at the superior temporal gyrus and frontal operculum by electroencephalography (EEG). The latest fMRI studies reported that the multivoxel activation patterns in the insula, thalamus and other regions could reliably discriminate between sour and other tastes (18 participants) [17] and that the discriminating activation patterns between different taste qualities in the insula were also affected by taste intensities (24 participants) [18]. These studies recruited between 6 and 24 participants, but recent neuroimaging guidelines have suggested that a sample size of approximately 30 participants is required for adequate power and to account for inter-subject variability [19,20,21]. Therefore, fMRI studies with a larger sample size (>30) would produce more representative data for the processing of sour taste, and enable an expansion of our knowledge about flavour, that is, the interaction of sour taste with congruent smell in the human brain.

Smell has two main divisions: orthonasal and retronasal smell [22,23,24]. Humans sense orthonasal and retronasal smells through the nostrils, and oral cavity and nasopharynx, respectively. Both types of smell activate the primary olfactory cortex, which includes the olfactory tubercle and piriform cortex; the secondary olfactory cortex, such as the hippocampus, OFC, insula, and operculum may also be involved [22,24,25,26,27,28]. Although both orthonasal and retronasal smell can activate the brain similarly when presented alone, humans react differently to the two types of smell when taste is added. Small et al. [29] have reported reduced cerebral blood flow (neural suppression) when an orthonasal smell was simultaneously presented with taste. In contrast, the retronasal smell is unconsciously used during flavour perception such as drinking tea [30] and activates the human brain during simultaneous presentation with taste [31,32,33,34,35]. Therefore, retronasal smell was chosen for the investigation of flavour perception in this study.

Several studies have reported that flavour stimulation involves the anterior insula, operculum, ventrolateral prefrontal cortex, and OFC [31,32,33]. In particular, the anterior insula is a site of convergence for multisensory information in flavour perception [31,33]. The posterior region of the frontal operculum (termed the rolandic operculum which is located around the central sulcus) plays a central role in flavour percept formation [33]. Based on these findings, we hypothesised that the communication between the anterior insula and rolandic operculum in the human brain is crucial for integrating taste and smell to form flavour perception.

The above-mentioned hypothesis can be tested by dynamic causal modelling (DCM), a hypothesis-driven statistical technique that determines effective connectivity within a brain network by analysing functional neuroimaging data [36]. This approach has been used in previous studies to investigate brain networks related to taste processing [37,38,39,40].

Taste and smell constitute the two major elements of flavour perception during food and drink intake; therefore, we defined flavour as the combined perception of taste and smell arising from the oral cavity in this study. We investigated the brain activity in response to each unisensory modality (taste or retronasal smell alone), and the combined multisensory effect (taste and smell presented simultaneously) using fMRI to elucidate the neuroscience of flavour perception. In addition, we sought to verify the structure of the flavour-processing network, including the causal relationship of activity between various brain regions and the location of taste–smell interactions, by DCM analysis. Using DCM analysis, we aimed to elucidate which brain structures within the modelled flavour network were the first to process flavour information, and determine the locations where taste and smell information modulated inter-node communication between brain structures. We hypothesised that neural taste and smell signals would be integrated at anterior insula and rolandic operculum.

## 2. Materials and Methods

### 2.1. Participants

We recruited 37 healthy, young, right-handed adults to participate in this study. All participants signed a written consent form. The exclusion criteria were smoking habit, taste and smell disorder, mental disorder, concurrent medication, claustrophobia, pregnancy, and any metal implant in the head and neck area that could interfere with fMRI. Participants were requested to have sufficient rest and fast for 2 h prior to fMRI scanning. The study complied with the Declaration of Helsinki for Medical Research involving Human Subjects, and was approved by the Institutional Review Board of The University of Hong Kong/Hospital Authority Hong Kong West Cluster (HKU/HA HKW IRB, UW 15-114). One participant withdrew before the fMRI due to an uncomfortable feeling induced by the sour tastant during the training session (described in section Training outside the MRI unit). Another participant withdrew because of an uncomfortable feeling in the MRI machine. This resulted in a total of 35 participants who completed this study (16 male and 19 female, aged 18–27 years, BMI 16.8–29.4 kg/m^2^).

### 2.2. Stimulus Solutions and Delivery

The stimuli included a sour tastant (0.025 M citric acid), mango odorant (1.5% by volume mango extract), and the flavour (0.025 M citric acid and 1.5% by volume mango extract). The stimuli concentrations were carefully chosen based on a pilot test among the research team members to ensure that the sour tastant did not cause irritation, and that the mango odorant presented a tasteless retronasal smell. The citric acid was in 0.025M, as more diluted solution (e.g., 0.01 M) presented a weak taste among research team members, whereas more concentrated solution (e.g., 0.05 M) was irritating to the tongue. The concentration of mango extract at 1.5% was the best—obvious smell without mango taste. Research team members pilot tested at 1% but the smell was very weak, and 3% had some mango taste. During the pilot study, lemon extract and mango extract were both tested, two locally common sour fruits with distinctive smells, but the former kept the taste sensation even at a very low concentration by volume. Thus, mango extract was chosen to represent the congruent odour in the experiment. Research assistants listed in the Acknowledgments tested mango extract together, and perceived predominantly sour smell without relating to sweet, fruity, woody and spicy smells. 

The sour taste–mango smell interaction in brain processing has not been investigated. Note that congruency is the association of the related stimuli (in this study, taste and smell) that affects the strength of the perceptual interaction [41]. The quality of taste and smell stimuli need to be in harmony with each other to give an effective flavour perception in the brain [32,35]. Citric acid is the main acidic component in mango juice and its concentration is adjusted to enhance the flavour and shelf life [42,43]; and therefore the sour taste and the mango smell are a harmonic combination. Distilled water was used to dissolve all the solutions and was also used as the control solution. 

All stimuli and water were delivered at room temperature (23–25 °C) through a tailor-made intraoral device in an original standardised delivery system. The system consisted of an intraoral device connected with separate delivery tubes for different stimulants to avoid cross-contamination [38,44], flow meters to control the flow rate of solution, and a suction apparatus to remove the solutions and saliva induced by taste sequentially and to avoid swallowing. The suction apparatus also removed the smell from the mouth and oropharynx, as demonstrated during the pilot tests. The system was computer-controlled to allow automated solution delivery. The timing of the introduction of the solutions onto the tongue was synchronised with the MRI time series.

### 2.3. Experimental Procedures

#### 2.3.1. Training Outside the MRI Unit

At least 1 day before the fMRI day, participants were trained in a computed tomography room, where the environment was similar to the MRI room, to familiarise themselves with the experimental procedure [44]. Training objectives were to breathe with the intraoral device as instructed in a particular sequence in order to effectively sense the odorants through the retronasal route with the device during fMRI scanning. 

#### 2.3.2. Sensory Evaluation of Taste, Smell, and Flavour before fMRI Scanning

Three solutions (sour taste, mango smell, and flavour of sour taste plus mango smell) were presented separately to participants without disclosing the content immediately before the fMRI scanning. These solutions were the same as those used during the fMRI, but were presented in cups [45]. Participants were required to sense approximately 5 mL of the solutions individually with gentle exhalation and then to discard them by spitting. Distilled water was used to rinse the mouth (without swallowing) between evaluations of each solution. Ratings of each solution were made on a visual analogue scale based on intensity (from 0 for no sensation to 10 for the strongest imaginable intensity). 

#### 2.3.3. fMRI Study Design

**Figure 1:**
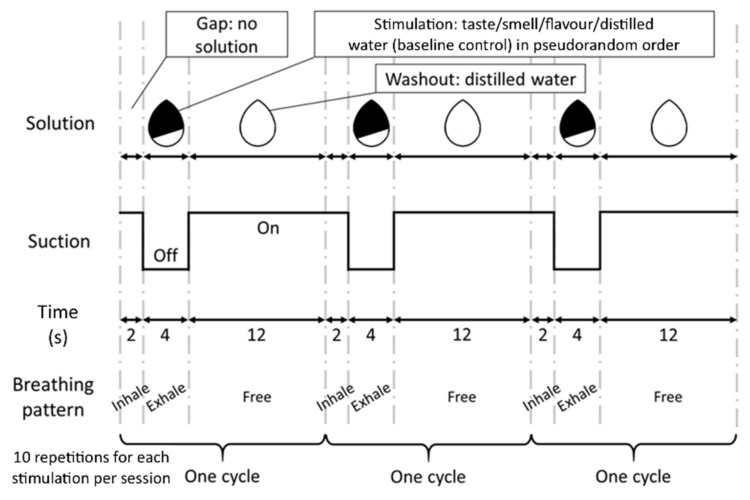
Experimental paradigm. The schematic diagram shows the three cycles of stimulation and washout, which were controlled by the delivery system. The first row represents the pattern of solution flow. The second row represents the period of suction operation. The third row represents the corresponding time for each period in seconds. The fourth row represents the instructed breathing pattern. During the first 2 s in each cycle, subjects did not receive any solution and were required to inhale. In the next 4 s, subjects were required to exhale gently and sense the stimulant (sour taste, mango smell, flavour of sour taste plus mango smell), or control (distilled water). These testing agents were delivered in pseudorandom order to avoid correct anticipation that might confound the brain activity signals. Afterward, subjects were free to breathe in the washout period. This washout period lasted for 12 s until subjects felt no solution being received, which indicated the start of the next cycle. Each stimulant and control were presented 10 times per session, resulting in a total of 30 repetitions per stimulus over the three fMRI sessions.

A block design was used in the fMRI sessions. The whole cycle of stimulation consisted of a gap without solution for 2 s, followed by stimulation for 4 s, and then a washout for 12 s (Figure 1). The 2 s gap was used to cue the participants to breathe gently (which was practiced during training) and remove any residual solution and smell from the previous cycle. During the 4 s stimulation period, participants were required to gently exhale with their nose and sense the solution. The suction pump was turned off during this period to minimise the disturbance on volatiles diffusing from the oral cavity to the olfactory receptors. The control and three experimental stimuli were pseudo-randomly delivered at a constant flow rate (1.25 mL/s) to give a volume (5 mL in total) that was sufficient to stimulate both the taste buds on the tongue and the olfactory receptors in the nasal cavity. Subsequently, distilled water was delivered for washout and the suction was turned on again. Each cycle of stimuli and control was repeated 10 times per session. With an administration of distilled water for 18 s at the beginning and 12 s at the end (total 30 s), each fMRI session lasted 12.5 min. Participants wore eye masks and earplugs throughout the experiment to avoid distraction by the external environment.

#### 2.3.4. fMRI Data Acquisition

Images were acquired with a 3-Tesla MRI scanner (Philips Achieva 3.0 System; Philips Medical System, Eindhoven, The Netherlands). There were 3 sessions of functional imaging followed by a 9.5-min session of anatomical imaging, giving a total scanning duration of 47 min. The functional images were collected by a T2*-weighted echo-planar imaging (EPI) sequence (repetition time, TR: 2000 ms, echo time, TE: 30 ms, slice number: 30, flip angle: 80°, field of view: 220 × 104.5 × 220 mm, matrix size: 80 × 80 pixels, voxel size: 3 × 3 × 3 mm^3^, slice thickness: 3 mm, slice gap: 0.5 mm, EPI factor: 73). The anatomical images were acquired using a T1-weighted magnetisation-prepared rapid gradient-echo (MPRAGE) sequence (TR: 6.9 ms, TE: 3.2 ms, flip angle: 8°, FOV: 250 × 250 × 155 mm, matrix size: 256 × 256 pixels, voxel size: 1 × 1 × 1 mm^3^, slice thickness: 1 mm).

### 2.4. Data Analysis

#### 2.4.1. Sensory Evaluation

Data from sensory evaluation of taste, smell, and flavour before fMRI scanning were analysed using SPSS 23.0 (IBM Corporation, New York, NY, USA). Shapiro–Wilk tests were performed to test the normality of the data distribution after excluding outliers (values greater than upper quartile plus 1.5 × interquartile range or less than lower quartile minus 1.5 × interquartile range). Paired *t* tests were performed to test the perceived intensity of flavour compared to taste and smell alone. 

#### 2.4.2. fMRI

##### fMRI Pre-Processing

Images were analysed using Statistical Parametric Mapping 8 (SPM8; Wellcome Institute of Cognitive Neurology, London, UK) implemented in MatLab R2011b (Mathworks Inc., Natick, MA, USA). The functional raw images were corrected with slice acquisition timing at 50% of TR. Next, these images were realigned to the mean image of each session to reduce movement artefacts. The anatomical raw image was coregistered with the mean functional images. Segmentation, bias correction, and spatial normalisation were then applied. All images were normalised to the Montréal Neurological Institute (MNI) space, and the functional images were smoothed with a full width at half-maximum isotropic Gaussian kernel of 8 mm.

##### Brain Mapping by Whole-Brain Analysis: Conventional General Linear Model Analysis

Task-specific effects at each voxel were estimated by a general linear model (GLM). Canonical haemodynamic response function was used to model the response. A high-pass filter with a cut-off of 128 s was applied to the data. Six movement realignment parameters (translations and rotations) were included as covariates of no interest to remove residual artefacts due to head movement. The stimulating periods were modelled as 3 conditions separately for each solution. The gap, washout, and the 30 s water administration were modelled as conditions of no interest. The control period was left unmodeled to act as the baseline [46].

The 3 conditions of interest were specified as 3 contrasts—‘sour taste’, ‘mango smell’, and ‘flavour’—at the first-level analysis to study brain activation for each participant. These contrasts were then used at the second-level analysis for the random effects analysis (one sample *t* test for this study) to test the consistency across participants. Voxels were considered significant at peak voxel *p* < 0.05, corrected for multiple comparisons using the family-wise error rate (FWE) or uncorrected *p* < 0.001 with minimum cluster size of 3 voxels in the predicted regions [24]. These *priori* regions for chemosensory perception of taste and smell included the insula and olfactory cortex, which are the primary cortices of taste and smell, respectively; and the operculum and prefrontal cortex, which comprise the secondary cortex and are involved in higher order responses in taste and smell stimulation [22,25,37,39,47]. Previous flavour studies have found that these regions are activated in response to their specific flavour, such as umami taste plus cooked chicken meat smell and sweet taste plus vanilla smell [31,32,33,35]. 

##### Effective Connectivity Analysis Using Dynamic Causal Modelling

Effective connectivity can be defined as the influence that a neural system exerts over another [48]. It is different from functional connectivity, which shows the temporal correlations between remote neurophysiological events [48]. In other words, effective connectivity expresses the causal functional relationships among brain regions that are the elements of neural systems. 

DCM was used to investigate effective connectivity of the flavour-processing network and the modulation by taste and smell in the current study. It estimates the causal functional relationship by a Bayesian approach and uses 3 key parameters to create a model space (a priori definition of hypothesis set and a collection of models possibly explaining effective connectivity): (1) intrinsic connections, which are the base of linkages and network among the volumes of interest (VOIs) in a brain; (2) driving input, which is applied to a brain region(s) to drive the system; and (3) modulatory input, which affects the strength of the connections. DCM employs a forward model, whereby output data are estimated using these parameter inputs and compared with the observed data. Bayesian model selection (BMS) was performed to determine the most plausible hypothesis (model structure) that described effective connectivity from the observed data among the participants.

##### Definition and Extraction of Volume of Interest

**Figure 2:**
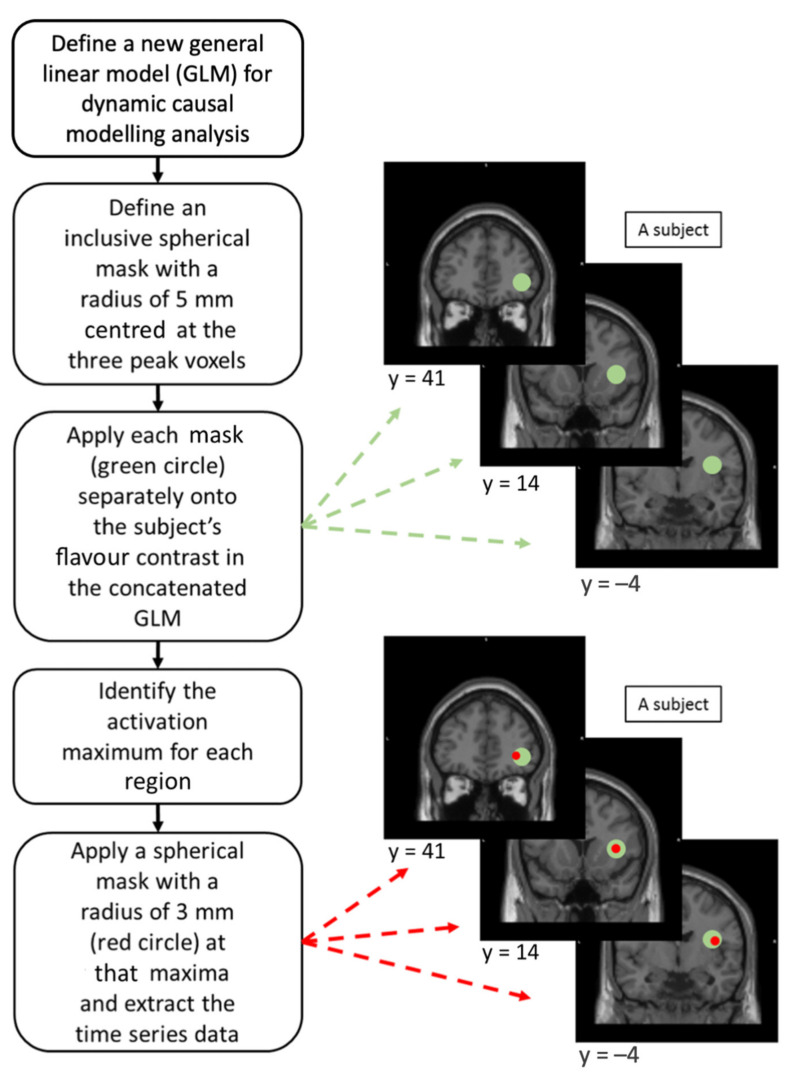
Illustrations on the procedures of volume of interest (VOI) extractions in dynamic causal modelling (DCM) analysis for individual subjects.

VOI represents the portion of brain structures from which data are extracted for analysis in DCM. Based on previous literature, we assumed that the insula, operculum, and ventrolateral frontal cortex (including the middle and inferior frontal cortex, and the OFC) are key sites for flavour perception [31,32,33,35]. We performed a region of interest (ROI) analysis on these 3 regions in the right hemisphere in the flavour contrast to localise their activation peaks (with FWE correction) for DCM analysis. These ROIs were defined by Wake Forest University Pickatlas tool [49]. Three activation peaks at the anterior insula, rolandic operculum, and inferior frontal triangularis (IFT) were identified and chosen for DCM analysis (Figure 2). The anterior insula and rolandic operculum were chosen because of their roles in flavour perception [31,33]. The IFT was chosen to represent higher-order processing for flavour perception because it has been reported as a secondary taste cortex [39], is associated with flavour perception [35], and is functionally connected with the OFC [50]. 

A new GLM was reconstructed for each participant by concatenating the 3 fMRI sessions with modification of session effect, high-pass filter, and autoregressive, AR(1), model to avoid deformation of the time series data [51]. Three spherical inclusive masks with a radius of 5 mm centred at those activation peaks were applied independently on each participant’s flavour contrast in their own concatenated, unthresholded GLM [37,38]. This was to find the maxima within each ROI and to ensure that the maxima among participants were at most 10 mm away from one another [37]. A spherical VOI with a radius of 3 mm was defined at each local maxima, corrected with the effect of interest, to extract the time series data for DCM analysis [51] (Figure 2). The 3 VOIs constituted the model system of the flavour-processing network.

##### Definition of Intrinsic Connections

**Figure 3:**
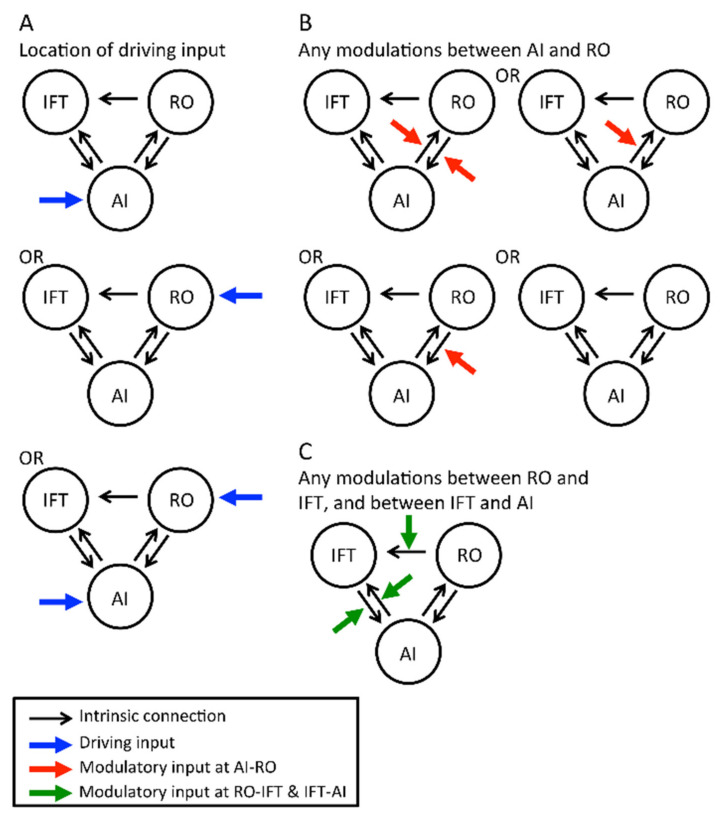
Composition of dynamic causal modelling (DCM) models. (**A**) Three possible options of sensory inputs (blue arrows), from the anterior insula (AI), rolandic operculum (RO), or both. (**B**) For each scenario of sensory input, there were four possible options of neural modulation by taste and smell signals (red arrows) of intrinsic connections between AI and RO. Therefore, there existed 12 groups of models (3 × 4), or in DCM terminology, 12 families. These 12 families were compared against each other. (**C**) In each family, there were eight models, representing any of the combinations of neural modulation at other sites (green arrows) which may either be present or absent (that is, there were two possibilities between RO and IFT, and four possibilities between AI and IFT). For how these intrinsic connections, driving input, and modulatory inputs were chosen based on existing literature, please see main text “Definition of driving inputs, Definition of modulatory inputs, and Inference on model structure using BMS” and Table 1.

Previous literature has shown bidirectional anatomical projections and functional connectivity between the insula and operculum [33,52], and the insula and IFT [52,53]; therefore, we defined a bidirectional intrinsic connection in these linkages (Figure 3A). Since we could only find one publication showing connections from the frontal/rolandic operculum to the IFT [39], only the intrinsic connection with this direction was included for these 2 regions (Figure 3).

##### Definition of Driving Inputs

The effect of flavour was defined as the driving input to initiate the flavour-processing network. As the anterior insula may be a convergent site and act as a detector to initiate the convergent process [31,33], we hypothesised that the anterior insula would be the location of the driving input. However, the rolandic operculum may also participate in the formation of the flavour percept [33]. Thus, 3 possible driving inputs (at the anterior insula, rolandic operculum, or both regions) were tested (Figure 3A).

##### Definition of Modulatory Inputs

Previous non-imaging studies have shown that the taste–smell interaction when perceiving a flavour depends on the original quality of taste and smell [54,55,56]. It is possible to have a specific brain region or connection that processes the information of taste and smell for their integration. Thus, we defined the brain responses to taste and smell as two independent modulatory inputs coexisting at the tested intrinsic connections. 

The anterior insula and rolandic operculum are highly involved in flavour perception, as mentioned above. The connections between these two regions are most likely involved in taste–smell integration. Thus, we hypothesised that taste and smell would modulate either one or both connections (Figure 3B). In addition, as the IFT is associated with flavour perception [31,35], the connections between the anterior insula and IFT were tested with the modulatory inputs. The modulation of the connection from the rolandic operculum to the IFT was also tested for completeness (Figure 3C).

##### Inference on Model Structure Using Bayesian Model Selection (BMS)

As we were interested in the model structure of the flavour-processing network, inference was made at both the model and family level using BMS [39,57]. There were in total 96 models in the model space for each participant (2^5^ × 3 = 96). Assuming the participants had the same model structure of effective connectivity, fixed effects (ffx) BMS was used to determine the optimal model with the highest posterior probability [57]. 

In addition, we confirmed the optimal driving and modulatory input region between the anterior insula and rolandic operculum by performing an ffx BMS at the family level [37]. All 96 models were first divided into 3 groups according to the location of driving input (Table 1, first column; Figure 3A), hence a total of 12 families (Table 1, Figure 3A,B). In each of these 12 groups, 8 models were present, representing combinations of any of the modulatory inputs between the rolandic operculum and IFT, and between the IFT and anterior insula (Figure 3C).

## 3. Results

### 3.1. Sensory Evaluation of Taste, Smell, and Flavour before fMRI 

The mean intensities of sour taste, mango smell, and flavour were 5.19 ± 1.72, 2.67 ± 1.46, and 6.67 ± 1.49, respectively. The mean intensity of the flavour was significantly higher than that of the sour taste and mango smell (*p* < 0.001, Bonferroni corrected). 

### 3.2. fMRI Results

#### 3.2.1. Brain Response to Taste, Smell, and Flavour 

**Figure 4:**
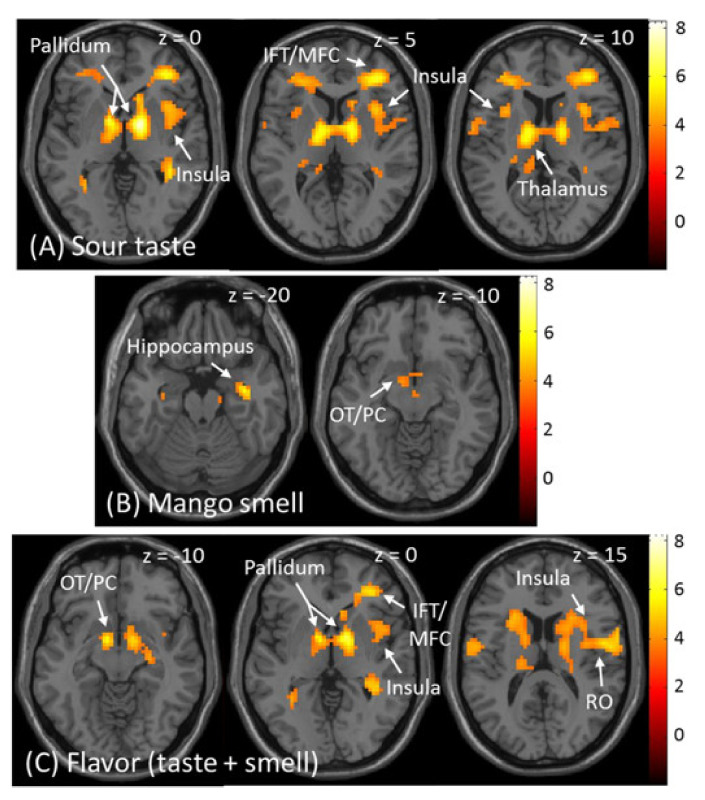
Brain responses in the group-level whole-brain analysis. (**A**) Activated areas by sour taste. (**B**) Activated areas by mango smell. (**C**) Activated areas by the flavour of sour taste plus mango smell. IFT: inferior frontal triangularis; MFC: middle frontal cortex; OT: olfactory tubercle; PC: piriform cortex; RO: rolandic operculum.

The fMRI results of the stimulation by taste, smell, and flavour from the group level random effects analysis are shown in Table 2. Significant activations for sour taste were observed in the pre- and postcentral gyrus, pallidum, prefrontal cortex, thalamus, cingulate cortex, middle temporal gyrus, and cerebellum (*p* < 0.05, FWE corrected). The middle and anterior insula were also activated, as predicted (*p* < 0.001, uncorrected) (Figure 4A). Significant activation for mango smell was observed in the hippocampus extending to the amygdala (*p* < 0.05, FWE corrected), and the olfactory tubercle extending to the piriform cortex and pallidum, as predicted (*p* < 0.001, uncorrected) (Figure 4B). Regarding flavour, significant activation was observed in the pre- and postcentral gyrus, pallidum, olfactory tubercle, prefrontal gyrus, cerebellum, and cingulate cortex (*p* < 0.05, FWE corrected). The insula and rolandic operculum (*p* < 0.001, uncorrected) were also activated by flavour, as predicted (Figure 4C).

#### 3.2.2. Effective Connectivity from DCM Analysis

**Figure 5:**
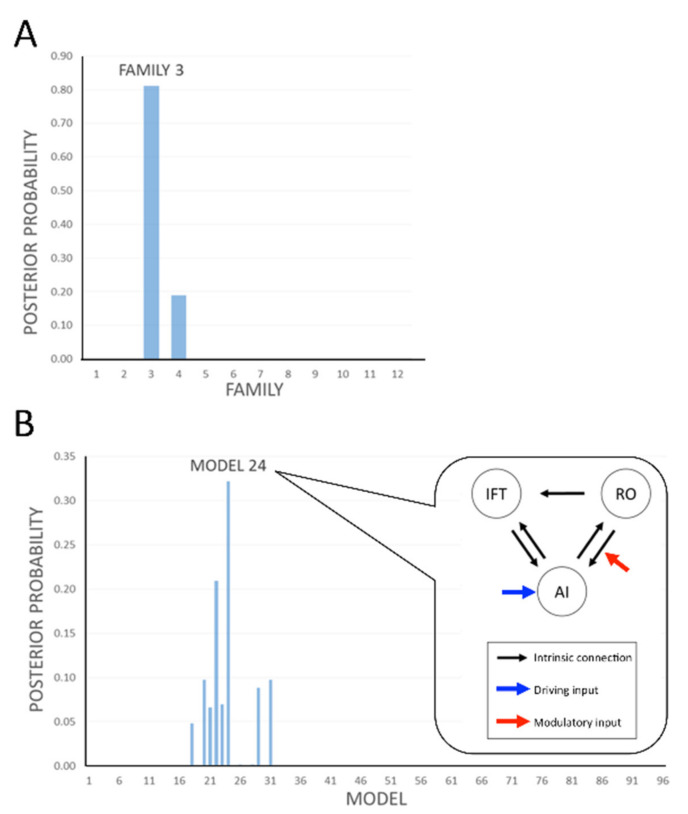
Results of the dynamic causal modelling (DCM) analysis of the flavour network modulated by taste and smell. We compared the results from our data under 96 different models per subject. (**A**) We grouped these into 12 families and pooled the results within each family. A comparison of the families, under the prior assumption that all families were equally likely, gave 81% probability that Family 3 provided the best explanation [that is, models with input at AI which all have modulation observable from RO to AI (Table 1)] for the dataset overall and 18% probability that Family 4 provided the best explanation for the dataset. Considering models within Family 4, the analysis indicated that input at AI has 100% posterior probability (Table 1, first row). (**B**) Within Family 3, model 24 appears to be the best model with 33% posterior probability, suggesting that the main modulation is from RO to AI. AI: anterior insula; RO: rolandic operculum; IFT: inferior frontal triangularis.

We compared the results from our data under 96 different models per subject. We grouped these into 12 families and pooled the results within each family. A comparison of the families, under the prior assumption that all families were equally likely, gave 81% probability that Family 3 provided the best explanation [models with input at AI which all had modulation observable from RO to AI (Figure 5A, Table 1)] for the dataset overall and 19% probability that Family 4 provided the best explanation for the dataset. Considering models within Family 4, the analysis indicated that input at AI had 100% posterior probability (Table 1, first row).

Within Family 3, model 24 (Figure 5B) appeared to be the best model with 33% posterior probability, suggesting modulation in RO to AI only. All remaining models in Family 3 indicated that regardless of other possible variations of modulation possibilities between RO to IFT and/or IFT to/from AI (Figure 3C), modulation from RO to AI remained key (total 81% posterior probability, Figure 5A).

## 4. Discussion

The current study reported the human brain response to a sour taste, mango smell, and the combined flavour. We have identified that the flavour-processing network response was initiated at the anterior insula and preferentially modulated by taste and smell at the connection from the rolandic operculum to the anterior insula. This suggested that there is a causal functional relationship between the rolandic operculum and anterior insula during flavour perception.

### 4.1. Sensory Evaluation of Taste, Smell, and Flavour

The mean intensity of flavour was significantly greater than for each unisensory solution. Our results are supported by previous findings, which have shown that combined flavour is more intense than taste or smell alone [31,33,56]. Odours used in those studies are vary such as retronasal smell of chicken, strawberry [31], ortho- and retronasal smell of orange juice [33], orthonasal smell of chest nuts, lychee, etc. [56]. Meanwhile, a previous study found no difference in intensity and hedonic ratings of odours administered orthonasally versus provided retronasally using 27 food-grade-quality candies [58]. 

Readers should be aware that, under the simplified flavour model used in this study, several factors contributing to flavour (except taste and smell) were omitted. Examples of these factors included trigeminal somatosensory perception, thermal perception, aftertaste and nociception [59,60]. Trigeminal somatosensory perception might be of particular relevance in this study during the tasting of citric acid. No participant reported unexpected feelings during tasting, such as tingling, stinging, or burning. It implied that the influence of trigeminal somatosensory perception was minimal in this study.

Furthers, there are other disturbing factors that may affect flavour perception, such as assimilation effect (or expectation) [61], mental imagery of the taste and/or smell [62,63], how the smell is described (named) to the participants [64], and level of expertise in olfaction [10,65]. For a comprehensive scheme of how flavour perception could be divided into three tiers of “what”, “reward/affective value”, and “decision-making/output”, please see Figure 1 of Rolls (2016) [66]. During the training phase, the participants in this study were informed that they would be receiving sour taste and mango smell. During the test phase, they were instructed to focus on the tasks. None of them worked in the food science field. Therefore, the participants should be relatively homogeneous in terms of these factors.

### 4.2. Brain Activation Maps for Taste, Smell, and Flavour

Results from the current study showed activation in some predicted brain regions with a p threshold that was uncorrected, such as activation in the insula by the sour taste; hippocampus, olfactory tubercle, and pallidum by the mango smell; and insula and rolandic operculum by the combined sour taste and mango smell flavour stimulation. Brain activation by taste and smell is typically smaller in magnitude compared with other sensations, such as vision; therefore, using an uncorrected p threshold may provide a better balance between false negatives and false positives, while still revealing meaningful results [24,67]. This implied that while our sample size was larger than the recommended sample size of ≥30 [19,20,21], a bigger sample size would provide greater power to detect brain activation.

### 4.3. Sour Taste

To the best of our knowledge, this is the first fMRI study to investigate the brain response to sour taste in >30 participants. Significant activation attributed to the sour taste was found in the middle and anterior dorsal insula. The middle insula is the primary taste cortex for salty, umami, and sweet tastants [37,38,68,69,70,71,72]. Activation of the middle insula in this study suggested that it is the primary taste cortex for identifying the sour taste. In contrast, previous studies have suggested that the anterior insula is associated with attention to taste detection or other taste-related tasks [40,73,74]. Therefore, the function of the anterior dorsal insula associated with activation following sour taste stimulation may be related to attention and active detection of the sour taste. 

### 4.4. Mango Smell 

The retronasal smell of mango activated the olfactory tubercle, which extended to the piriform cortex, pallidum, and hippocampus. The olfactory tubercle and piriform cortex represent the primary cortex for smell, whereas the striatum (including the ventral pallidum and hippocampus) is the secondary cortex for smell [25]. We found activation of the olfactory tubercle and piriform cortex, which is consistent with past olfactory studies that report a significant response to odour detection in these regions [22,24,26,75]. The activation of the pallidum suggests a role in food-related reward processes [24,76,77,78,79]. The role of the hippocampus during olfactory perception is unclear; however, it is commonly activated in other studies investigating brain activation with olfaction. In addition, it is connected with the primary olfactory cortex [22,24,25,80,81]. The activated hippocampus may be related to odour memory because of its role in the human memory system [82,83,84]. Further studies are needed to elucidate the function of the hippocampus in the olfactory system.

### 4.5. Combined Flavour of Sour Taste and Mango Smell

This is the first study to investigate brain activation induced by a combination of sour taste and mango smell. Our results showed that the anterior and middle insula, rolandic operculum, and IFT were activated by ‘flavour’. The anterior insula may be a convergent site for flavours [31,33,35] and also activated by food odour [1]. Therefore, we propose that the anterior insula would be involved in the convergence of sour taste and mango smell. The middle insula is the primary taste cortex [37,38,68,69,70,71,72] and may be involved in the primary identification of sour taste in flavour. Further, a study using orange juice, which is a sweet and sour taste mixed with orange smell, reports activation of the posterior part of the frontal operculum (rolandic operculum) is associated with in the formation of a flavour perception [33]. The rolandic operculum may support the same function for a flavour with a pure sour taste. Moreover, different flavour studies show activation of the lateral prefrontal cortex; however, its function has not been confirmed [31,35]. One recent study has suggested this region is a secondary taste cortex receiving inputs from the insula and frontal/rolandic operculum [39]. The IFT activation in the current study may also reflect higher-order processing for chemosensory perception. This finding was also consistent to Sinding et al. [85] in which beef stock odour-induced saltiness enhancement was reported to occur in high-level integration areas; but different to Onuma et al. [86] in which soy sauce odour-induced saltiness enhancement occurred in central gustatory processing. Meanwhile, data from an event-related potential study suggested that taste–smell integration might be initiated at an early stage of the central pathway [87]. Finally, a prior meta-analysis showed that food odour could also activate the amygdala and precentral gyrus [1].

To the best of our knowledge, this is the first study to show significant activation in the olfactory tubercle following flavour presentation. Seubert et al. [33] is the only study among the few studies on flavour perception that has shown evidence of the involvement of the temporal piriform cortex following joint activation of smell and flavour. The results in this study have shown more direct evidence showing the olfactory response to flavour stimulation in the human brain. Nevertheless, one limitation of this study is that real mangoes are both sour and sweet. It was reported that citrus was a congruent combination with sweet and sour [88]. Therefore, the current research does not fully reflect the stimulation associated with consumption of mango. Further, due to technical limitations, we did not evaluate the compliance/discrepancies of the respiratory sequences of the participants. Another limitation is that the neural responses of our participants in certain brain areas were relatively weaker with uncorrected statistics. However, our results using uncorrected statistics remains useful from predicted regions with a pre-defined minimum cluster size [24]. This study was conducted on only 35 participants. Future studies should recruit more participants to increase the power to validate the findings.

### 4.6. Effective Connectivity in the Flavour-Processing Network

DCM analysis showed model 24 in Family 3 was the best fit model. The flavour triggered the flavour-processing network through the anterior insula only (Figure 5). Previous studies characterise the anterior insula as having a convergent ability for taste and smell [31,33,89]. The primary taste and olfactory cortices are connected to the anterior insula independently and directly [25,52,90]. Taken together, the current result suggested that taste and smell converge at the anterior insula, which drives flavour perception. 

In addition, we have presented novel evidence showing that taste and smell modulate the connection from the rolandic operculum to the anterior insula. The exact mechanism of signal transduction underpinning such modulation, for example through opioid receptors that control taste perception and nutrient intake [91], remains to be elucidated. The modulation from taste and smell in this connection may reflect the dependency on the original taste and smell during flavour perception [54,55,56]. This modulation supports previous findings that taste and smell are integrated at both the rolandic operculum and anterior insula [31,33,35]. Indeed, functional connectivity between the rolandic operculum and anterior insula during flavour perception has been previously reported [33]. Our study using DCM has suggested a causal functional relationship during flavour perception. Our results suggest that the integration of a sour taste and mango smell first occurs at the rolandic operculum followed by the anterior insula.

## 5. Conclusions

We have shown the communications in the human brain responding to a sour taste, mango smell, and combined flavour of both for a larger volunteer force. We identified that the flavour-processing network was initiated at the anterior insula and preferably modulated by taste and smell at the connection from the rolandic operculum to the anterior insula. This suggests a causal functional relationship between the rolandic operculum and anterior insula during flavour perception. These findings illustrate the usefulness of DCM to advance our understanding of chemosensory perception and neural mechanisms underlying flavour perception in the brain. The current findings allow a better understanding of how neural signals triggered by sour taste and mango smell presented in liquid form interact in the human brain, which may in turn assist our understanding of sour food appreciation. Future studies with a bigger sample size are appreciated to confirm neural signal interactions of sour taste and other congruent smells. 

Humans integrate multiple senses on a daily basis, and it is very important to study how these senses work together. Current limitations in methodology have led to studies that encode/decode a single sense in many human perception studies. This study constructed a novel methodology to study the integration and synergy of taste and smell. This enables studies to decipher influences on human behaviour from ‘flavour/senses’, which may underpin disease risk. Hence, these studies, including the current research, may be relevant to medicine, in particular for patients with brain lesions such as stroke or following head and neck surgery, who are undergoing rehabilitation. Understanding any deficits or difficulties with sensory perception may help to retain quality of life. Furthermore, the current study provided important objective data that clarify flavour processing in the brain and the correlation with simple sensory evaluation in the mouth, which are very useful for the food industry.

## Figures and Tables

**Table 1:** Families designated according to the location of the driving input and modulatory inputs between AI and RO ^a^ in the DCM analysis.

	Modulatory Input (s) ^c^			
Location of Driving Input ^b^	both AI 🡪 RO and RO 🡪 AI	AI 🡪 RO	RO 🡪 AI	None
AI	1 (M1–8)	2 (M9–16)	3 (M17–24)	4 (M25–32)
RO	5 (M33–40)	6 (M41–48)	7 (M49–56)	8 (M57–64)
Both AI and RO	9 (M65–72)	10 (M73–80)	11 (M81–88)	12 (M89–96)

AI: anterior insula, RO: rolandic operculum, and M: model. ^a^ Modulatory inputs between RO and IFT could be present or absent, and those between AI and IFT could be bidirectional, unidirectional (either way), or absent. Thus, there were a total of 2 × 2 × 2 = 8 models (M) for each family (1–12). ^b^ Please refer to Figure 3A. ^c^ Please refer to Figure 3B.

**Table 2:** Activation peaks from stimulation by taste, smell, and flavour in the group-level whole-brain analysis.

Location	Cluster Size (Voxel)	MNI Coordinate (x,y,z)	*t*	*p*-Value ^a^
**Sour taste**				
R Precentral gyrus	4463	48,−13,34	8.23	<0.001
R Postcentral gyrus	-	54,−7,25	7.60	<0.001
R Pallidum	-	15,−1,1	7.52	<0.001
L Precentral gyrus	-	−48,−13,37	7.03	<0.001
L Pallidum	-	−12,2,1	6.26	0.003
R Inferior frontal triangularis/Middle frontal gyrus	-	36,41,4	6.00	0.006
L Thalamus	-	−12,−7,10	5.88	0.007
L Middle cingulate cortex	-	−6,−25,28	5.64	0.013
R Superior medial frontal gyrus/Anterior cingulate cortex	-	15,14,34	5.59	0.015
R Middle cingulate cortex	-	9,−22,31	5.57	0.016
L Anterior cingulate cortex	-	−9,26,22	5.42	0.023
R Supramarginal gyrus	-	30,−40,31	5.23	0.037
R Middle temporal gyrus	-	39,−40,1	5.17	0.043
L Superior medial frontal gyrus	-	−9,11,37	5.13	0.047
L Anterior insula	-	−30,14,13	4.97	<0.001 ^b^
R Anterior insula	-	36,14,7	4.70	<0.001 ^b^
R Anterior insula	-	39,11,1	4.47	<0.001 ^b^
R Middle insula	-	42,8,−2	4.45	<0.001 ^b^
R Cerebellum	565	12,−58,−23	6.70	0.001
Vermis	-	0,−43,−20	6.12	0.004
R Vermis	-	3,−52,−26	6.10	0.004
L Cerebellum	-	−15,−58,−23	5.89	0.007
Vermis	-	0,−67,−17	5.18	0.042
**Mango smell**				
R Hippocampus	55	36,−10,−20	5.59	0.014
R Hippocampus	-	39,−16,−17	5.04	<0.001 ^b^
L Olfactory tubercle/Piriform	109	−9,−4,−11	4.57	<0.001 ^b^
R Pallidum	-	12,5,−2	4.36	<0.001 ^b^
R Pallidum	-	12,−1,−2	4.16	<0.001 ^b^
**Flavour (sour taste plus mango smell)**				
R Precentral gyrus	2331	48,−10,34	7.05	<0.001
R Precentral gyrus	-	60,2,28	6.42	0.002
R Pallidum	-	12,2,1	6.29	0.002
L Pallidum	-	−12,2,−2	5.90	0.006
L Olfactory tubercle/Piriform	-	−9,−1,−8	5.42	0.021
R Superior medial frontal gyrus	-	6,2,64	5.12	0.042
R Anterior insula	-	33,14,13	4.95	<0.001 ^b^
R Rolandic operculum	-	39,−4,19	4.70	<0.001 ^b^
R Middle insula	-	39,−1,4	3.92	<0.001 ^b^
R Cerebellum	425	12,−58,−23	6.82	0.001
L Cerebellum	-	−12,−61,−23	6.34	0.002
L Postcentral gyrus	310	−48,−16,31	6.26	0.003
L Postcentral gyrus	-	−57,−13,28	6.06	0.004
L Middle cingulate gyrus	190	−3,−25,28	5.55	0.015
R Inferior frontal triangularis/Middle frontal gyrus	127	33,41,4	5.28	0.029

L: Left, R: Right. ^a^ Unless otherwise specified, the peak voxels were reported at *p* < 0.05 (FWE corrected). ^b^ Uncorrected with a minimum cluster size of 3 voxels in predicted regions [24].

## Additional Information

This paper was created in part base upon a thesis submitted to HKU for a MPhil degree of J.L.K.S. with the following bibliometric details and citation. Suen, L. (2016). Chemosensory perception in human brain: the interaction of taste and smell as a simplified flavour model. (Thesis). University of Hong Kong, Pokfulam, Hong Kong SAR, China.
